# Identifying Human SIRT1 Substrates by Integrating Heterogeneous Information from Various Sources

**DOI:** 10.1038/s41598-017-04847-7

**Published:** 2017-07-04

**Authors:** Zichao Zhai, Ming Tang, Yue Yang, Ming Lu, Wei-Guo Zhu, Tingting Li

**Affiliations:** 1 Department of Biomedical Informatics, School of Basic Medical Sciences, Peking University Health Science Center, Beijing, 100191 China; 2 Key Laboratory of Carcinogenesis and Translational Research (Ministry of Education); State Key Laboratory of Natural and Biomimetic Drugs; Beijing Key Laboratory of Protein Posttranslational Modifications and Cell Function; Department of Biochemistry and Molecular Biology, School of Basic Medical Sciences, Peking University Health Science Center, Beijing, 100191 China; 3 Peking-Tsinghua University Center for Life Science; Peking University, Beijing, 100191 China; 4 School of Medicine; Shenzhen University, Shenzhen, 518060 China; 5 Institute of Systems Biomedicine, School of Basic Medical Sciences, Peking University Health Science Center, Beijing, 100191 China

## Abstract

Most proteins undergo different kinds of modification after translation. Protein acetylation is one of the most crucial post-translational modifications, which causes direct or indirect impact on various biological activities *in vivo*. As a member of Class III HDACs, SIRT1 was the closest one to the yeast sir2 and drew most attention, while a small number of known SIRT1 substrates caused difficulties to clarify its function. In this work, we designed a novel computational method to screen SIRT1 substrates based on manually collected data and Support Vector Machines (SVMs). Unlike other approaches, we took both primary sequence and protein functional features into consideration. Through integrating functional features, the Matthews correlation coefficient (MCC) for the prediction increased from 0.10 to 0.65. The prediction results were verified by independent dataset and biological experiments. The validation results demostrated that our classifier could effectively identify SIRT1 substrates and filter appropriate candidates for further research. Furthermore, we provide online tool to support SIRT1 substrates prediction, which is freely available at http://bioinfo.bjmu.edu.cn/huac/.

## Introduction

Post-translational modifications (PTMs) are a general phenomenon occurred among all species and known to be essential for regulating diverse functions and dynamically coordinating signaling networks^1^. There is a variety of different types of PTMs, such as acetylation, ubiquitination, methylation and phosphorylation^2^. Among these modifications, acetylation is the process of transferring an acetyl functional group from one molecule to another, and deacetylation is the reverse reaction of acetylation. These two processes are mediated by histone acetyltranferases (HATs) and histone deacetylases (HDACs)^3^. Based on their homology to yeast proteins, the eighteen histone deacetylase are divided into four groups. Class I, II, IV includes eleven classical Zn^+^-dependent HDACs, while class III HDACs’ function depend on NAD^+^ as a cofactor^4, 5^. In mammals, the class III HDACs contains Sirtuin family from silent mating type information regulator 2 homolog 1 (SIRT1) to SIRT7. Among them, SIRT1, homolog to the yeast Sir2 protein, is a member of the mammalian sirtuin protein (SIRT1-7) family and has diverse significant roles in various biological processes that encompass genomic stability, tumorigenesis, energy metabolism and cellular stress resistance^6^. SIRT1 can deacetylate a variety of substrates which extends biological functions. As reported, SIRT1 is shown to be able to deacetylate histones and preferentially deacetylate H3K9^7^ and H4K16^8^ *in vitro*, interacts and deacetylates H1K26 and mediates heterochromatin formation^9^. In addition to histone substrates, more and more non-histone proteins had been reported to serve as SIRT1 substrates. For example, SIRT1 regulates genomic stability by deacetylating the DNA damage repair related proteins such as Ku70^10^, NBS1 (Nijmegen breakage syndrome 1)^11^, Werner syndrome protein (WRN)^12^. SIRT1 modulates gluconeogenesis in the liver through deacetylation of some important factors such as CRTC2 and FOXO1^13^. In addition, SIRT1 regulates immune system though deacetylation of NF-κB^14^ and FOXP3^15^. All sorts of information remind us the importance and necessity of conducting research on SIRT1 and its deacetylation substrates.

As we know, enzymes affect biological process through interacting with substrates, and clarifying substrate specificity is the fundamental step to develop methods for finding SIRT1 substrates. While using oriented peptide libraries containing acetylated lysine, Blander *et al*. found that substrates of SIRT1 have no obvious motif specificity^16^. In 2012, based on Stable Isotope Labeling by Amino acids in Cell culture (SILAC), SIRT1 Wild-type and Knockout cells using HPLC-MS/MS analysis, were applied to proteomics for identification and quantification of lysine acetylation, which revealed SIRT1-response Lys acetylome and deacetylation related cellular pathways^17, 18^. This method was effective to screen SIRT1 substrates, but still time-consuming and expensive. Bioinformatics prediction method is a feasible approach to filter SIRT1 substrates.

In this study, we developed a SIRT1 substrate prediction method based on the manually collected substrate sites of SIRT1. Through integrating heterogeneous biological information from various sources, we constructed the SIRT1 substrate classifier based on Support Vector Machines^19^. Then, SIRT1 substrates were filtered from known acetylated peptides and 5,684 putative substrate sites from 1,630 proteins were obtained. Finally, we evaluated the efficiency of our prediction method through comparing with independent dataset and biological experiments.

## Results

### Sequence feature analysis and prediction performance with only sequence feature

**Figure 1:**
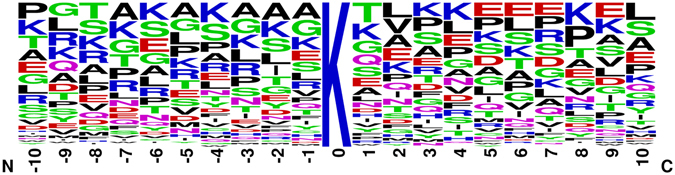
Frequency of amino acids in each position of SIRT1 substrates. The height of symbols within the stack indicates the relative frequency of each amino at that position.

Previous studies revealed two opposite opinions about whether substrates of SIRT1 have sequence specificity. One opinion is that substrates of SIRT1 share little sequence specificity^16^. In contrasting reports, the residues proximal to the SIRT1 deacetylated lysine sites are crucial to modifications^20, 21^. Here, we used WebLogo^22^ to visualize amino acids frequency in each position of SIRT1 substrates. After trimming 129 manually collected lysine centered SIRT1 substrate peptides to 21 amino acids length, 118 substrate peptides from 51 proteins were remained for subsequent analysis (Supplementary Table S1). From the amino acids distribution of these 118 peptides, it seems that there is no significant motif recognized by SIRT1, although there are some positions existing a little amino acid preference. As shown in Fig. 1, lysine (K) and alanine (A) are abundant from −7 to −1 near the N-terminus. Near the C-terminus, threonine (T) is most likely to be recognized at position 1. Except position 8, glutamic (E) is preferred from 5 to 9.

Then we further estimated the prediction performance merely based on primary sequence. To test the prediction performance with different sequence length, the compiled 118 SIRT1 substrate sequences of 51 proteins were trimmed to 11, 13, 15, 17, 19, and 21 amino acids length, which were still lysine centered and treated as positive datasets. To avoid exaggeration in prediction accuracy, we reduced the redundancy of sequence similarity between peptides. So, peptides with more than 70% sequence similarities in the positive samples left only one. Negative datasets, whose sample size and sequence length are the same as corresponding positive datasets, were randomly selected from the background set and redundancy of sequence similarity was avoided at the same time. The background set is composed of the whole lysine centered sites in human proteome after excluding proteins in positive datasets (extracted from Swiss-Prot database, version Release 20150323). Then, 4/5 positive and negative sample peptides were taken as training set. Meanwhile, the remaining 1/5 positive and negative sample peptides were taken as testing set. The above test processes were performed 100 times and negative datasets would be re-selected in each time of test. The final performance was the average of those 100 tests.

**Figure 2:**
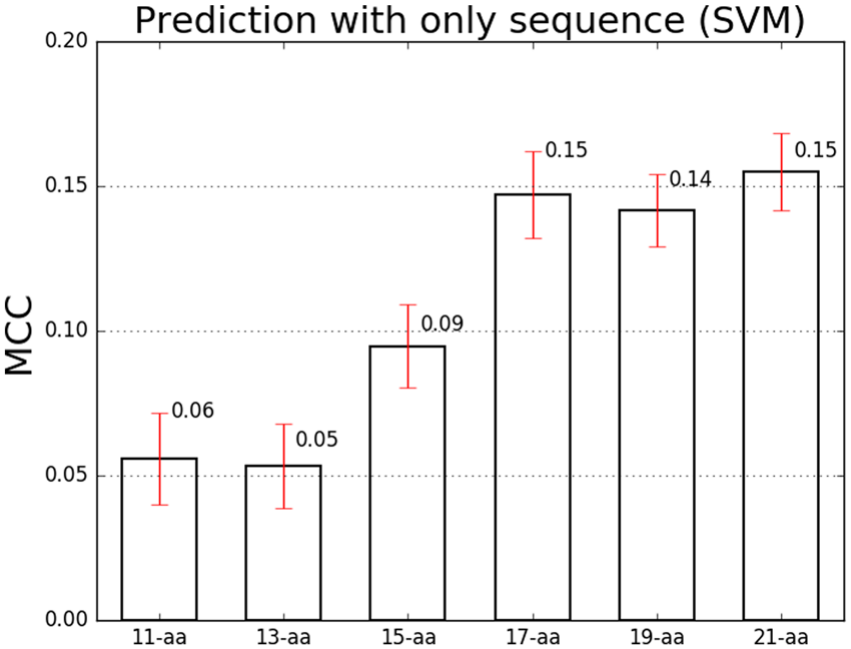
Prediction performance of SIRT1 substrates with different sequence lengths based on SVM. Error bar charts containing mean value and standard error were utilized here to visualise MCC results.

As the prediction performance shown in Table 1, we found sensitivity (Sn) varied from 41% to 48% and specificity (Sp) varied from 57% to 73%. In Fig. 2, the MCC of all sequence length performed not well (from 0.06 to 0.15) and the best performance came from 21 amino acids sequence length. We also used Random Forests and Neural Networks to construct SIRT1 substrate prediction models with sequence features, while their performance were even worse than SVM (Supplementary Table S2). The above analysis indicated the poor performance of identifying SIRT1 substrates with only sequence features.

**Table 1:** Sn and Sp of prediction with different sequence length (SVM).

Seq Length	Sensitivity	Specificity
11-aa	48%	57%
13-aa	48%	57%
15-aa	44%	65%
17-aa	43%	70%
19-aa	42%	71%
21-aa	41%	73%

### Prediction performance with sequence and functional features

Unideal prediction performance with only sequence reminded us that protein functional features might be useful in improving the prediction performance. Thus, we collected the functional annotations from following databases: (1) Biological Process (BP), (2) Cellular Component (CC) and (3) Molecular Function (MF) annotations from the Gene Ontology (GO) database (version 20150313); (4) protein-protein interaction (PPI) information from the STRING database (version 9.1); (5) protein functional domain feature from the Pfam database (release 27.0). Then for each protein, a large number of functional annotations could be extracted as functional features from these five types of annotations. To reduce the dimensionality of the feature space, only the over- or under-represented functional annotations would be selected. Hypergeometric tests were utilized to detect over- or under-represented functional annotations based on SIRT1 substrate proteins in the positive set, with the human proteome downloaded from the Swiss-Prot database as a background. Terms with Bonferroni corrected *p*-values less than 1e-2 were considered as significant ones. If there were too many significant terms, top 100 significant terms with lowest *p*-values would be used. Then those selected functional annotations were used as functional features.

**Figure 3:**
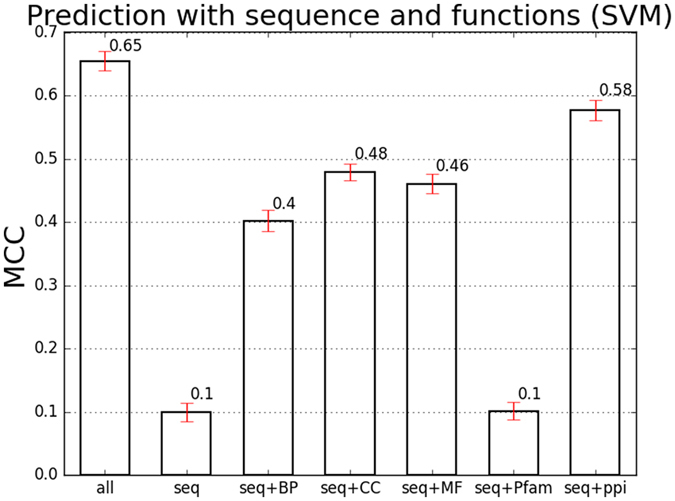
Prediction performance of SIRT1 substrates with sequence and functional features based on SVM. Error bar charts containing mean value and standard error were utilized here to visualise MCC results.

To estimate the prediction performance of integrating the above five types of functional features, and contributions brought by each type of functional features, seven feature combinations were obtained, as shown in Fig. 3. The test strategy was similar to that in the estimation of prediction performance with only sequence features. However, it has to be noted that two sites in one protein sharing same functional features, and if two substrate sites from one protein were separated into training set and testing set, it would cause an overestimation of prediction with functional features. So the positive and negative datasets were randomly divided into two parts (4/5 and 1/5) at protein level and then corresponding sites were extracted as the training and testing sets. In addition, the functional features were re-selected based on the 4/5 positive training samples in each time of 100 tests.

As shown in Fig. 3, compared with the prediction performance with only sequences, integrating all functional features increased the MCC from 0.10 to 0.65. The Sn and Sp of integrating all features were 0.71 and 0.92 (Table 2). Furthermore, from the prediction performance of integrating sequence features with different kinds of functional features, it is obvious that most of the functional features contribute to the improvement of the prediction performance except those from the Pfam database. Compared with sequence features alone, integrating the PPI information increased MCC from 0.10 to 0.58 (Fig. 3). BP, CC, and MF annotations brought improvement of MCC to 0.40, 0.48 and 0.46 respectively (Fig. 3). These results indicated that integration of functional features can greatly improve the prediction performance.

**Table 2:** Sn and Sp of prediction with sequence and functions (SVM).

Func Groups	Sensitivity	Specificity
**Seq**	53%	56%
+BP	53%	84%
+CC	70%	78%
+MF	60%	84%
+Pfam	52%	57%
+STRING	63%	92%
**+all**	71%	92%

### Building SIRT1 substrates classifier and application to the known human acetylation sites

We constructed the final prediction SVM models with all known positive samples and negative samples randomly selected from the background set. Significant functional features were enriched based on all positive samples (Supplementary Table S3). The sample size of the background set is much larger than that of positive samples. We found that the selection of different negative sample sets would influence the final prediction results. When we made predictions with classifier which include only one SVM model, the overlap of two prediction results is only 74.24%. To make the prediction results repeatable, we adopted the strategy of multiple models. Each SVM model was built with the same positive sets and different randomly selected negative sets, and the final prediction results were decided by their positive prediction votes. It means that we selected a suitable positive prediction vote as cutoff, and sites with positive prediction votes no less than the cutoff would be predicted as putative candidates. We found that when the classifier included 9 SVM models and 5 of 9 models return positive predictions as the cutoff, the overlap of two predictions reached 90.07%. So, the final classifier adopted 9 models.

**Figure 4:**
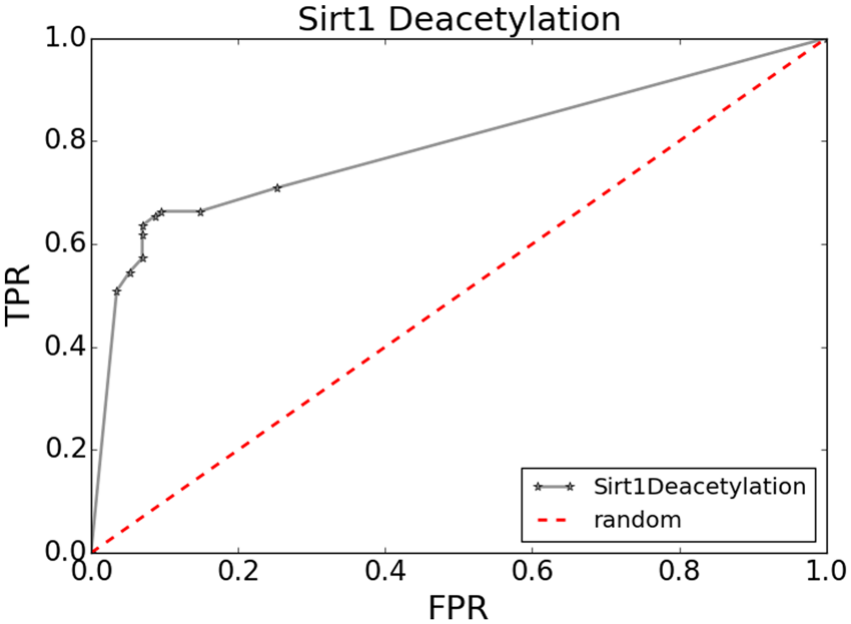
ROC curve of SIRT1 substrates prediction. Points on the curve were calculated with different vote number as cutoff. FPR: false positive rate, TPR: true positive rate.

Furthermore, we explored different influence from different vote number to prediction performance. With different vote number as cutoffs, we obtained the points on ROC curve (Fig. 4) by the average performance of 100 rounds of evaluation tests. To balance the Sn and Sp, those candidates with no less than 5 of 9 models returning positive predictions were predicted as putative substrates of SIRT1. In addition, users can control the false positive rate by selecting more stringency cutoffs. For example, when we set the cutoff as 9, the Sn and Sp reached 51% and 97% respectively (Supplementary Table S4). Meanwhile, a web server (http://bioinfo.bjmu.edu.cn/huac/) was provided for the prediction of SIRT1 substrates to facilitate the users in related fields.

With the SIRT1 substrate classifier, we firstly screened putative SIRT1 substrates from human acetylation sites downloaded from the PhosphoSitePlus database. After excluding those acetylation sites of the known substrate proteins of SIRT1, 17,572 acetylation sites from 6,338 human proteins were obtained (Supplementary Table S5). With vote number 5 as the threshold, 5,412 sites from 1,590 proteins were predicted as the substrates of SIRT1 (Supplementary Table S6), whose proportion of sites is 30.80%. If our method can discriminate the substrates of SIRT1 from others, then we would expect the proportion of predicted substrates from the acetylation sites are higher than that from the random lysine sites. To test this hypothesis, we randomly selected 19,949 lysine sites from 10,050 proteins (Supplementary Table S7). As for the random set, 3,112 sites from 1,644 proteins were predicted to be the substrates of SIRT1 (Supplementary Table S8), whose proportion of sites is 15.60%. The proportion of predicted substrates generated by the acetylation sites was significantly higher than that generated by the random lysine sites (*p*-value < 2.2e-16 by Pearson’s Chi-squared test with Yates’ continuity correction). With vote number 9 as the threshold, the proportion of predicted substrates from the acetylation sites were still significantly higher than that from the random lysine sites (23.55% VS 10.76%, *p*-value < 2.2e-16 by Pearson’s Chi-squared test with Yates’ continuity correction). These results indicated that our method could discriminate the substrates of SIRT1 from others.

### Performance evaluation with independent dataset and experimental validation

**Figure 5:**
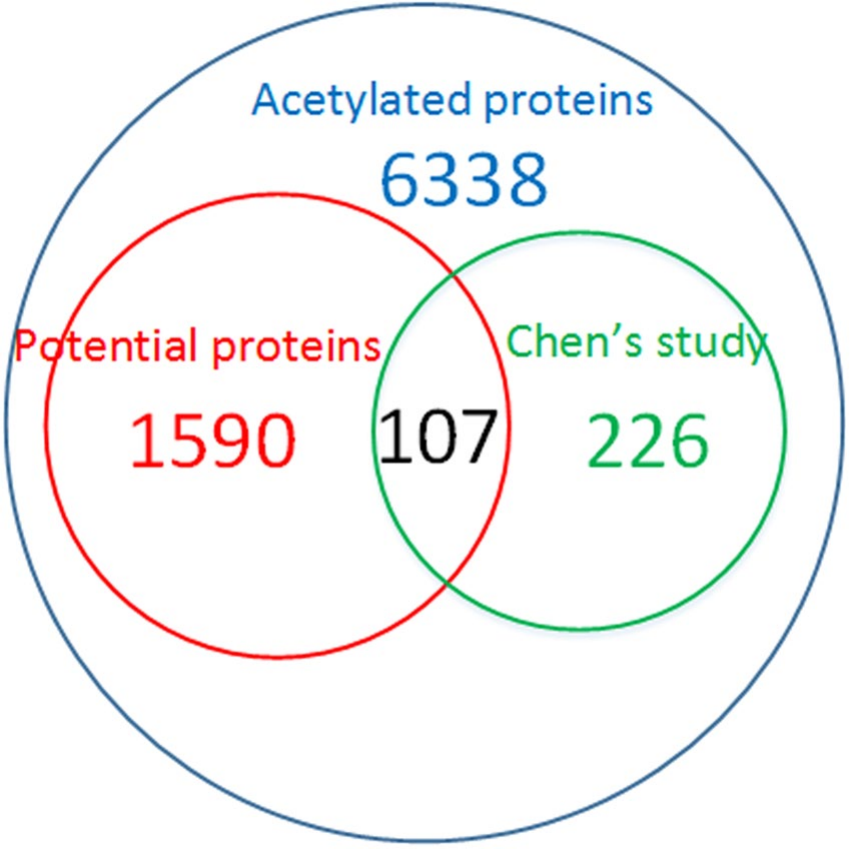
Overlap between our prediction results and Chen’s study.

In a previous study, a group of putative SIRT1 substrate sites in mouse were generated by Chen *et al*.^18^. Totally 270 SIRT1 substrate proteins that contained lysine sites exhibiting 2-fold change of acetylation level between WT and KO cells were collected (Supplementary Table S9). Those 270 proteins were taken as the putative substrate proteins of SIRT1. After ortholog mapping of those mouse proteins to human by BioMart and excluding those proteins in our positive set, 260 proteins were left. Of those 260 putative SIRT1 substrate proteins by Chen *et al*., 226 were in the list of the 6,338 human acetylation proteins. Though those 226 putative substrates might contain some indirect substrates of SIRT1, we still expected a significant proportion of those 226 proteins can be classified as the substrates of SIRT1 by our method. As shown in Fig. 5, the overlap between the 1,590 predicted SIRT1 substrates by our method (with the vote number 5 as the cutoff) and the 226 putative SIRT1 substrates by Chen *et al*. is 107 (*p*-value < 7.138e-15 by Pearson’s Chi-squared test with Yates’ continuity correction), which revealed that our method is effective in filtering SIRT1 substrates.

**Figure 6:**
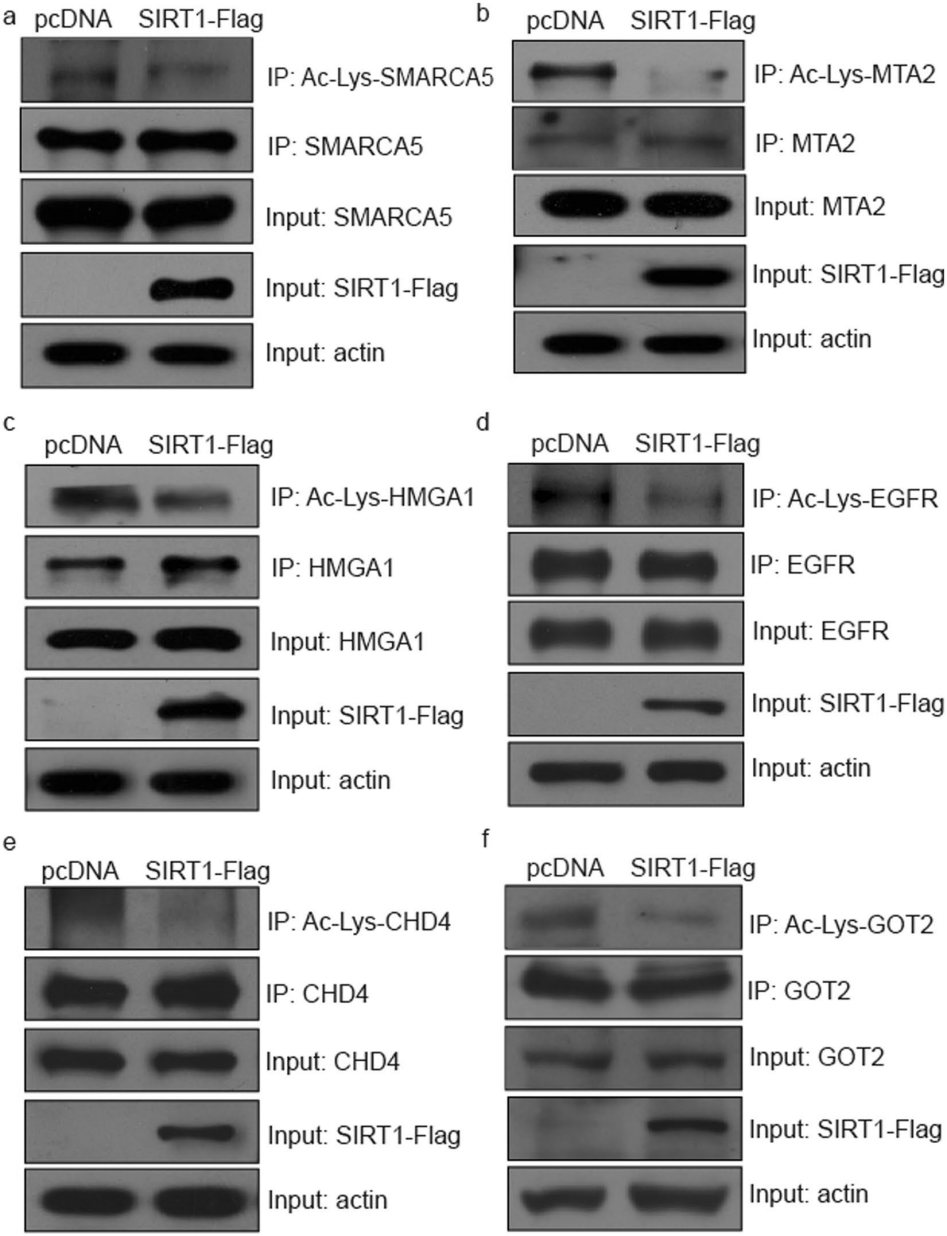
(**a**–**f**) Detection of predicted deacetylation substrates by SIRT1 with immunoprecipitation and Western blotting. Acetylation levels of SMARCA5 (**a**), MTA2 (**b**), HMGA1 (**c**), EGFR (**d**), CHD4 (**e**) and GOT2 (**f**) decreased after SIRT1 overexpression. pcDNA: pcDNA-vector transfection. SIRT1-Flag: Flag-tagged SIRT1 plasmid transfection as indicated in the figure. Equal amounts of indicated proteins were immunoprecipitated, followed by western blotting with pan-lysine acetylation antibody, used to detect the acetylation of immunoprecipitated proteins.

Besides compared with other study, we designed biological assays to confirm the prediction results. According to the laboratory condition and the prediction results, we selected 9 proteins with highest voting scores (9 votes), which included SMARCA5 (SWI/SNF related, matrix associated, actin dependent regulator of chromatin, subfamily a, member 5), MTA2 (metastasis associated 1 family, member 2), HMGA1 (high mobility group AT-hook 1), EGFR (epidermal growth factor receptor), CHD4 (chromodomain helicase DNA binding protein 4), GOT2 (glutamic-oxaloacetic transaminase 2), CDK6 (cyclin dependent kinase 6), FANCD2 (Fanconi anemia complementation group D2) and MSH6 (mutS homolog 6). The acetylation status of these 9 proteins were monitored after overexpression of SIRT1. When SIRT1-Flag tagged expression plasmid was transfected into Human colon cancer HCT116 cell line, the acetylation levels of SMARCA5, MTA2, HMGA1, EGFR, CHD4 and GOT2 decreased as equal amounts of the proteins were immunoprecipitated (Fig. 6). The other three proteins CDK6, FANCD2 and MSH6 did not exhibit any change, at least under the condition of overexpression of SIRT1 (see Supplementary Fig. S1). For the proteins that could not be deacetylated by the way of overexpressing SIRT1 plasmid, there might be several reasons. First, these proteins really could not be deacetylated by SIRT1, which means they were not SIRT1 substrates. Second, these proteins could be deacetylated by SIRT1 but not in the cell line we used. Third, because of the feature of different proteins, the deacetylation by SIRT1 were not detected under the experimental state we built. In addition, sometimes deacetylation happened under specific cell signal stimulation, for example, DNA damage treatment or growth factors. The overexpression of Class III HDACs might be insufficient to induce deacetylation in some conditions. In order to exclude the possibility of non-specific deacetylation, 7 acetylated proteins predicted not to be substrates were tested. These 7 acetylated proteins ACSL1 (acyl-CoA synthetase long-chain family member 1), USP47 (ubiquitin specific peptidase 47), OTUB1 (OTU deubiquitinase, ubiquitin aldehyde binding 1), KDM2A (lysine demethylase 2A), JADE2 (jade family PHD finger 2), FABP5 (fatty acid binding protein 5), INTS3 (integrator complex subunit 3) were selected from the lists with score 0, the results were consistent with our predictions that the acetylation levels of these 7 acetylated proteins did not show any change after SIRT1 overexpression (see Supplementary Fig. S2).

## Discussion

In this study, we proposed a computational method to filter substrates of SIRT1 though integrating sequence features and various functional features. Primary sequence feature has been widely used to predict PTM substrates. But some types of PTMs do not have significant sequence specificity, like the substrates of SIRT1 in this study. Poor performance of prediction with only sequence features demonstrated that classifiers with sequence features alone are insufficient to recognize SIRT1 substrates (Supplementary Table S2). The performances of prediction based on SVM gained significant improvement after integrating functional features with sequence features (Fig. 3). We also tested the prediction performance of Random Forests and Neural Networks after integrating functional features with sequence feature. We found that with either Random Forests or Neural Networks, the prediction performance could be improved greatly (Supplementary Table S10), and Random Forests even performs better than SVM. These results demonstrated that no matter what kind of classifiers were adopted, the benefit of combining functional information with primary sequences in the prediction of SIRT1 substrates was obvious. In addition, since there is no software available for SIRT1 substrate prediction, comparisons with bioinformatics approaches for identifying substrates of other acetylation enzymes were also conducted. We made prediction of SIRT1 substrates with the ASEB (Acetylation Set Enrichment Based method)^23^ and GPS-PAIL (GPS Prediction of Acetylation on International Lysines)^24^ methods and comparing the prediction performance based on Sn, Sp and MCC (details of the experimental processes can be found in the Supplementary Information). The Sn, Sp and MCC of ASEB were 0.19, 0.92 and 0.1498. The Sn, Sp and MCC of GPS-PAIL were 0.13, 0.92 and 0.0730. Both of these two methods made prediction based on primary sequence information and their prediction performance were much worse than our new method, whose Sn, Sp and MCC were 0.71, 0.92 and 0.6549. All these results confirmed the benefit of combining functional information with primary sequences in predicting SIRT1 substrates.

Among the five types of functional information we used, PPI brought the most significant improvement to prediction performance (Fig. 3). As shown in Supplementary Table S3, 626 proteins were found to be significantly over-represented with *p*-value cutoff 0.01, which means that a significant number of SIRT1 substrates interact with these 626 proteins. The protein with the most significant *p*-value was SIRT1 itself, which is reasonable. The other top significant proteins include p300 and p53. It has been found that p300 cooperates with SIRT1 to regulate the histone H2K56 acetylation homeostasis *in vivo* ^25^. For p53, many papers revealed that SIRT1 exerted multiple effects on p53 by directly deacetylating p53 or managing other factors such as Tip60 which was deacetylated by SIRT1 and acetylates p53^26–28^. With those reasonable functional terms as prediction features, it is not surprising to improve the prediction performance greatly. We then screened SIRT1 substrates from the known acetylation sites and obtained a reasonable list of potential SIRT1 substrates.

Altogether, our method presented here provides novel and helpful insights for biologists to investigate the substrates of SIRT1, and should facilitate the cognition of SIRT1 deacetylation in health and disease.

## Materials and Methods

### Data preparation

We manually collected substrates of SIRT1 from published literature with key words, SIRT*[Title/Abstract] AND deacetylate*[Title/Abstract], in PubMed, and 2,470 papers were returned until April 9th, 2015. After reviewing, 129 SIRT1 substrate sites from 54 human proteins were obtained (Supplementary Table S1). For deacetylated sites, we compiled their peptides with central deacetylated lysine and surrounding 10 amino acids on both sides, 118 substrate peptides from 51 proteins were remained (Supplementary Table S1), which were taken as positive samples here. The source reference of each deacetylated sites can be found in Supplementary Table S1 and researchers can gain more details from the original articles. Negative samples were randomly selected from the background set.

### Sequence and functional features coding

Here, machine learning package scikit-learn^29^ (scikit-learn-0.16.1) was used to construct SVM models with the radial basis function (RBF) kernel. To code these functional features for SVM classifier, for those selected functional terms, if the proteins have the feature, the corresponding bit would be represented by 1, otherwise represented by 0. For the sequence features, we simply defined a 20-bit binary tuple for each amino acid, for instance, lysine (K) is represented by a 20-dimensional vector [0,0,0,0,0,0,0,0,0,1,0,0,0,0,0,0,0,0,0,0]^T^. Therefore, if the peptide length of candidate sequence is 21 and N functional features were enriched, the dimension of input vector is 20*21+N.

### Statistical parameters for evaluating

In our statistical analysis, we abbreviated True Positive, True Negative, False Positive and False Negative as *TP*, *TN*, *FP* and *FN*. Sn, Sp and MCC are defined as equations 1, 2 and 3:

1 $$Sn=\frac{TP}{TP+FN}$$

2 $$Sp=\frac{TN}{TN+FP}$$

3 $$MCC=\frac{(TP\times TN)-(FN\times FP)}{\sqrt{(TP+FN)\times (TN+FP)\times (TP+FP)\times (TN+FN)}}$$

### Cell culture and transfection

Human colon cancer cell line HCT116 used in this research were purchased from the American Type Culture Collection. The cells were cultured in McCoy’s 5A Medium supplemented with 10% foetal bovine serum (FBS) and 1% antibiotics. All cell lines were maintained in a humidified incubator at 37 °C with 5% CO2. One 100mm plate using lipotransfectamine 2000 (20 µl; Invitrogen) and plasmids (8 µg) to perform transfection. The transfected cells were then harvested at 48 h post-transfection.

### Co-immunoprecipitation and Western blot

Cell extracts were prepared by lysing cells in Nonidet P-40 buffer (50 mM Tris-HCl, pH 7.5, 300 mM NaCl, 1% Nonidet P-40, 0.1% SDS, 2 mM EDTA, 10 mM sodium butyrate, 1% cocktail(Roche, Basel, Switzerland)). SMARCA5, MTA2, HMGA1, EGFR, CHD4 and GOT2 were immunopurified from clarified supernatant with the indicated antibodies incubated at 4 °C overnight. Then, 30 μL of protein G or A Sepharose slurry (GE healthcare, NY, USA) was added and incubated for 2 h at 4 °C. The beads were washed by Nonidet P-40 buffer three times at 1,000 rpm 4 °C for 1 min. The precipitated components were analyzed by Western blotting as previously described with minor modifications^30^. Anti-pan-Lysine acetylation antibody was used to detect the acetylation of immunoprecipitated proteins.

## Electronic supplementary material

Supplementary information

Table S1

Table S2

Table S3

Table S5

Table S6

Table S7

Table S8

Table S9

Table S10
