# Supplementary material for: Identifying Human SIRT1 Substrates by Integrating Heterogeneous Information from Various Sources
Source: Sci Rep. 2017 Jul 4;7:4614. doi: 10.1038/s41598-017-04847-7 (PMC5496887; doi:10.1038/s41598-017-04847-7)
Supplement: Supplementary file 1 — Supplementary information [file 41598_2017_4847_MOESM1_ESM.pdf]

Identifying Human SIRT1 Substrates by Integrating Heterogeneous Information from Various Sources

Zichao Zhai[1,+], Ming Tang[2,+], Yue Yang[1], Ming Lu[1], Wei-Guo Zhu[2,3,4,*], and Tingting Li[1,5*]

Prediction based on GPS-PAIL and ASEB

GPS-PAIL (GPS Prediction of Acetylation on International Lysines)[1] and ASEB (Acetylation Enrichment Set Based method)[2] were bioinformatics approaches designed for identifying protein acetylation sites. To demonstrate the benefit of integrating functional features with primary sequence, performance test of predicting with these two approaches were carried out.

GPS-PAIL method mainly used BLOSUM62 to score potential substrates of acetylation enzymes depended on primary sequence information. For each query peptide sequence, after comparing the sequence similarities with those sequences in the positive dataset, sequences with high similarity scores will be predicted as potential substrates. The best scoring strategy was obtained based on positive and negative datasets. We repeated the prediction process of GPS-PAIL. The manually compiled 118 SIRT1 substrate sequences were trimmed to 21 amino acids length and treated as positive dataset. Negative dataset, whose sample size is also 118, was composed of lysine centered peptide sequences with corresponding length. Those negative samples were randomly selected from the whole human proteome (Swiss-Prot database, version Release 20150323) after excluding proteins in positive dataset. Then, 4/5 positive and negative sample peptides were taken as training set to explore the best scoring strategy. Meanwhile, the remaining 1/5 positive and negative sample peptides were taken as testing set. The above test processes were performed 100 times and negative datasets would be re-selected in each time of test. The final performance was the average of those 100 tests. For convenience of comparison with our new method, we used the cutoff which would generate Sp near 0.92 to calculate Sn and MCC.

ASEB also depended on primary sequence information to recognize potential substrate sites of acetylation enzymes, proposed by our team previously. For a query sequence, through comparing its similarities with sequences in the positive set and the background set, sequence with high similarity with samples in the positive dataset will obtain smaller p-value score. The compiled 118 SIRT1 substrate sites were taken as the positive set. The background set was composed of 10000 lysine centered peptide sequences which were randomly selected from the whole human proteome (Swiss-Prot database, version Release 20150323, excluding proteins in positive set). The five-fold cross-validation on positive samples was carried out to estimate Sn. Then, 118 negative peptide sequences were randomly selected from the whole human proteome to estimate Sp with all positive samples as training set. Samples in the negative dataset would be re-selected for 100 times and the final Sp was the average of these 100 tests. For MCC, TP and FN were got by the five-fold cross-validation on positive dataset; TN and FP were got by the average of prediction performances on 100 negative datasets. For convenience of comparison with our new method, we used the cutoff which would generate Sp near 0.92 to calculate corresponding TP, FN, TN, FP.

Supplementary table legends

Supplementary Table S1. SIRT1 substrates proteins and sites used as positive dataset for the prediction.
The highlighted lines are abandoned data which can't be trimmed into standard prediction uniform.

Supplementary Table S2. Prediction based on different classifiers (only sequence).
Machine learning package scikit-learn (scikit-learn-0.18) was used to construct models. For Random Forest, the tree number was set to 100. For Neural Networks, the neuron number of hidden layer was set to two times of neuron number of input layer plus one ($N_{hid}=2*N_{in}+1$). The test strategy and coding manners are the same with predictions based on SVM.

Supplementary Table S3. Enriched functional features of SIRT1 substrates proteins.
Enrichment terms were represented for deacetylation proteins from the GO, Pfam and STRING database.

Supplementary Table S4. Prediction performance of different cutoffs (SVM).

Supplementary Table S5. Human acetylation sites obtained from PhosphoSitePlus database.

Supplementary Table S6. Potential SIRT1 substrates filtered from human acetylation sites.
Every lysine site were predicted by nine models. Votes were calculated by the prediction result of each model. Lysine sites which were predicted positive by more than or equal to five models (score>=5) were considered as potential substrate sites.

Supplementary Table S7. Randomly selected lysine sites from the human proteome.

Supplementary Table S8. Potential SIRT1 substrates filtered from randomly selected lysine sites.
Every lysine site were predicted by nine models. Votes were calculated by the prediction result of each model. Lysine sites which were predicted positive by more than or equal to five models (score>=5) were considered as potential substrate sites.

Supplementary Table S9. SIRT1 substrates from Chen's study.
270 SIRT1 substrate proteins contained lysine sites exhibiting 2-fold change of acetylation level between WT and KO cells in Chen's study.

Supplementary Table S10. Prediction based on different classifiers (sequence and functions).
Machine learning package scikit-learn (scikit-learn-0.18) was used to construct models. For Random Forest, the tree number was set to 100. For Neural Networks, the neuron number of hidden layer was set to two times of neuron number of input layer plus one ($N_{hid}=2*N_{in}+1$). The test strategy and coding manners are the same with predictions based on SVM.

Supplementary figures

Supplementary Figure S1 a-c. Detection of acetylation levels to be SIRT1 deacetylation substrates but remain unchanged with immunoprecipitation and Western blotting.

Acetylation levels of CDK6 (a), FANCD2 (b), MSH6 (c) remain unchanged after SIRT1 overexpression. pcDNA: pcDNA-vector transfection. SIRT1-Flag: Flag-tagged SIRT1 plasmid transfection as indicated in the figure. Equal amounts of indicated proteins were immunoprecipitated, followed by western blotting with pan-lysine acetylation antibody, used to detect the acetylation of immunoprecipitated proteins.

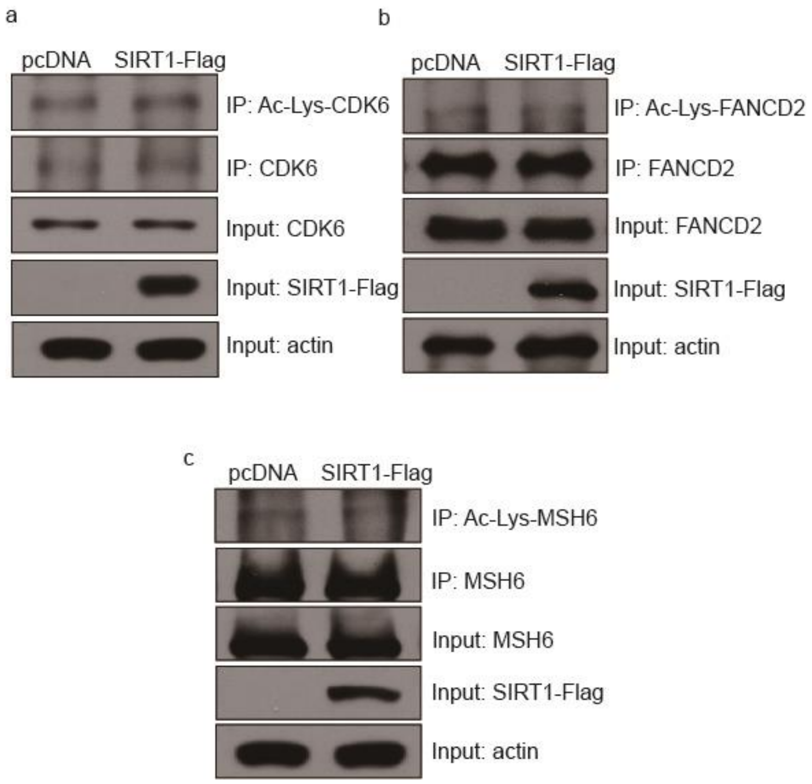

Supplementary Figure S2 a-g. Detection of acetylation levels not to be SIRT1 deacetylation substrates with immunoprecipitation and Western blotting.

Acetylation levels of ACSL1 (a), USP47 (b), OTUB1 (c), KDM2A (d), Jade2 (e), FABP5 (f) and INTS3 (g) remain unchanged after SIRT1 overexpression. pcDNA: pcDNA-vector transfection. SIRT1-Flag: Flag-tagged SIRT1 plasmid transfection as indicated in the figure. Equal amounts of indicated proteins were immunoprecipitated, followed by western blotting with pan-lysine acetylation antibody, used to detect the acetylation of immunoprecipitated proteins.

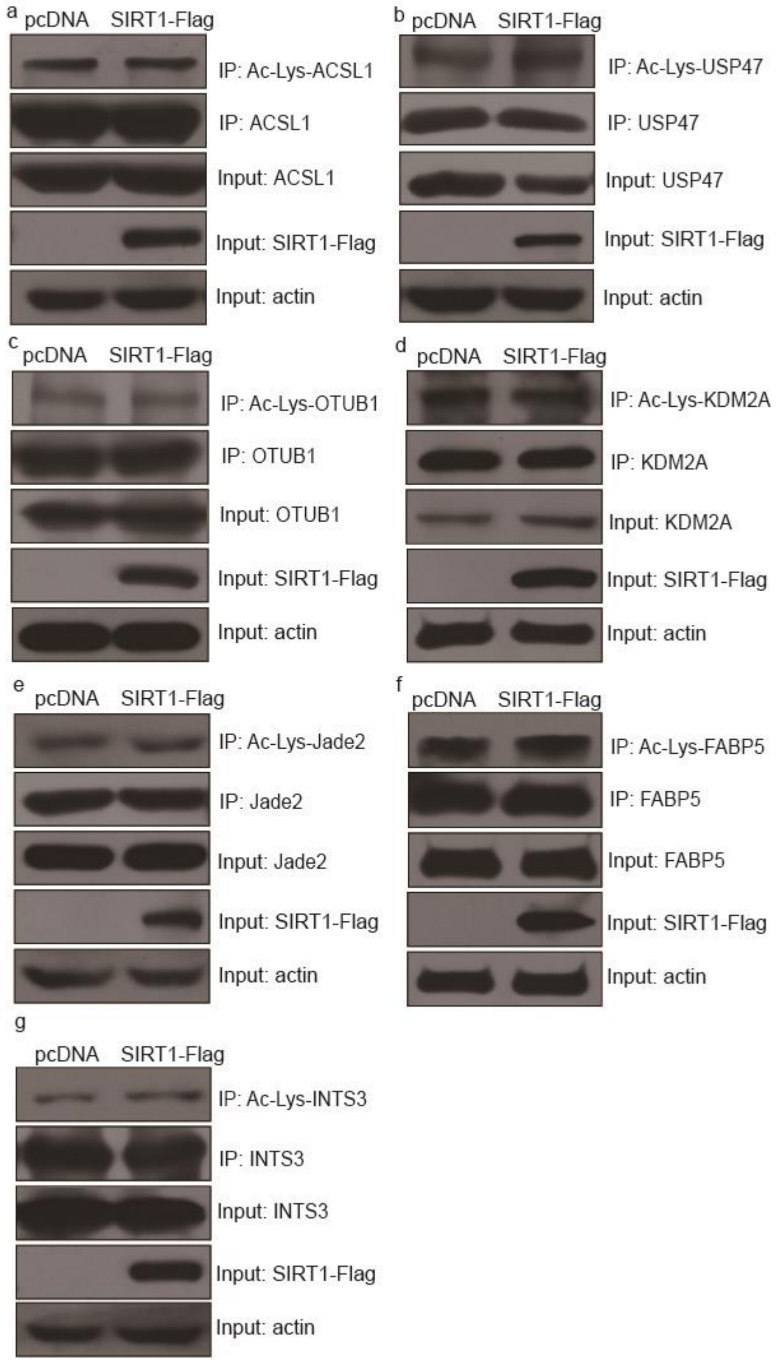

Supplementary tables

Supplementary Table S4. Prediction performance of different cutoffs (SVM).

Cutoffs (Votes)	Sensitivity	Specificity
9	51%	97%
8	55%	95%
7	57%	93%
6	62%	93%
5	64%	93%
4	65%	91%
3	66%	90%
2	66%	85%
1	71%	75%

Reference:

1 Deng, W. *et al.* GPS-PAIL: prediction of lysine acetyltransferase-specific modification sites from protein sequences. *Scientific reports* **6**, 39787, doi:10.1038/srep39787 (2016).

2 Wang, L., Du, Y., Lu, M. & Li, T. ASEB: a web server for KAT-specific acetylation site prediction. *Nucleic acids research* **40**, W376-379, doi:10.1093/nar/gks437 (2012).
